# Practical Bacteriology, Blood Work and Animal Parasitology

**Published:** 1911-03

**Authors:** 


**Practical Bacteriology, Blood Work and Animal Parasitology.** By **E. R. Stitt,** A.B., Ph.G., M.D., Surgeon U. S. Navy. Second edition. With 91 illustrations. 12 mo, pp. 345. Philadelphia: P. Blakiston's Son & Co. 1910. (Cloth, $1.50.)

When the Panama canal is finished and opened to the world, our obligations to preventive medicine will be more adequately understood and appreciated than ever before.

It seems more than appropriate that the medical corps of our navy should make a contribution to the rapidly increasing literature of bacteriology and we are greatly pleased with the book of Dr. Stitt. It is small, concise in statement and, as the author says, is "A manual of Laboratory and Clinical Diagnosis." It is a "little volume, a practical one," the design being "to keep the book within the limits of a pocket manual."

The scientific poise of the writer is evident throughout. We note with satisfaction his mention of "Diseases of unknown or not definitely determined etiology," and observe enumerated with others, "Epidemic Poliomyelitis, Measles, Scarlet Fever, Smallpox and Rabies."

Our readers will be pleased with the last word on an important subject from an eminent source—the American Navy.

